# WorMachine: machine learning-based phenotypic analysis tool for worms

**DOI:** 10.1186/s12915-017-0477-0

**Published:** 2018-01-16

**Authors:** Adam Hakim, Yael Mor, Itai Antoine Toker, Amir Levine, Moran Neuhof, Yishai Markovitz, Oded Rechavi

**Affiliations:** 1 Sagol School of Neuroscience, Tel Aviv University, Tel Aviv, Israel; 2 Department of Neurobiology, Wise Faculty of Life Sciences, Tel Aviv University, Tel Aviv, Israel; 3 Department of Biochemistry and Molecular Biology, Institute for Medical Research Israel-Canada (IMRIC), School of Medicine, The Hebrew University of Jerusalem, Jerusalem, Israel

**Keywords:** *Caenorhabditis elegans*, Machine learning, Deep learning, High-throughput image analysis, Feature extraction, Image processing, Phenotype analysis

## Abstract

**Background:**

*Caenorhabditis elegans* nematodes are powerful model organisms, yet quantification of visible phenotypes is still often labor-intensive, biased, and error-prone. We developed WorMachine, a three-step MATLAB-based image analysis software that allows (1) automated identification of *C. elegans* worms, (2) extraction of morphological features and quantification of fluorescent signals, and (3) machine learning techniques for high-level analysis.

**Results:**

We examined the power of WorMachine using five separate representative assays: supervised classification of binary-sex phenotype, scoring continuous-sexual phenotypes, quantifying the effects of two different RNA interference treatments, and measuring intracellular protein aggregation.

**Conclusions:**

WorMachine is suitable for analysis of a variety of biological questions and provides an accurate and reproducible analysis tool for measuring diverse phenotypes. It serves as a “quick and easy,” convenient, high-throughput, and automated solution for nematode research.

**Electronic supplementary material:**

The online version of this article (doi:10.1186/s12915-017-0477-0) contains supplementary material, which is available to authorized users.

## Background

*Caenorhabditis elegans* nematodes are powerful genetic model organisms which are instrumental for research on a wide range of biological questions. It is relatively simple to grow *C. elegans* under tightly regulated environmental conditions, and since the worm has a short generation time (3 days) and a large brood size (±250), large sample sizes and statistical power are easily obtained. In many cases, however, when phenotypic features are examined, the advantage of having a large sample size comes with great cost, because of the need to manually analyze the features of interest in the tested animals. Programs for quantifying *C. elegans*’ phenotypes from still images exist, for example, WormSizer [1], Fiji [2], QuantWorm [3], and WormToolbox [4]. However, the analysis process of these programs is not fully automated, and not all informative phenotypic features can be analyzed simultaneously in one package. Moreover, each type of software answers different specific research requirements.

We created WorMachine as a fast, friendly, and high-throughput image processing platform. WorMachine enables automated calculation of many morphological and Fluorescent features and accessible machine learning techniques for higher level features-based analysis (described in detail in the Implementation and Methods sections), such as classification and phenotype scoring. WorMachine is entirely MATLAB-based and combines the capabilities of different programs into one software package; the user-friendly interface was designed to suit investigators with no background in MATLAB, image processing, or machine learning, and it requires no additional plugins or installations. WorMachine is not limited to any specific image format, resolution, acquisition software, or microscope.

### Implementation

**Fig. 1:**
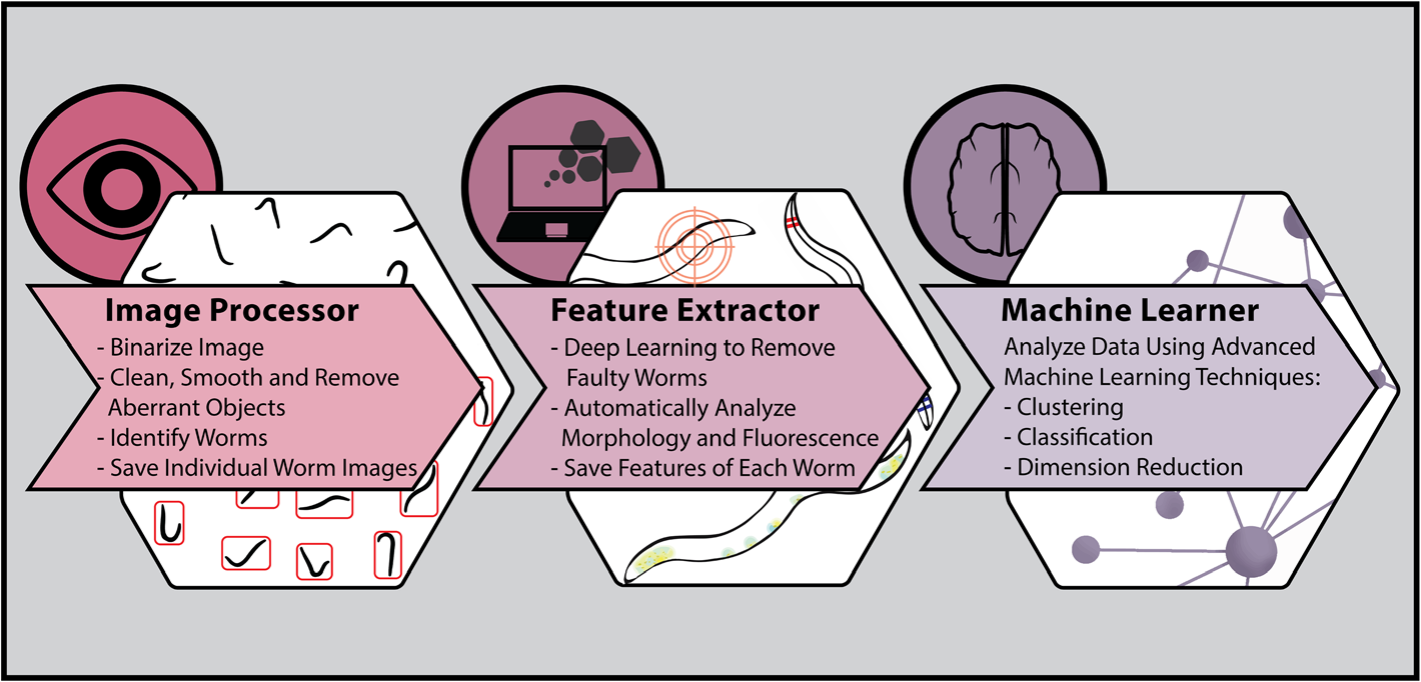
WorMachine workflow. The software includes three sequential programs: an Image Processor, a Feature Extractor, and a Machine Learner

WorMachine’s workflow includes three sequential programs: Image Processor, Feature Extractor, and Machine Learner (Fig. 1). WorMachine’s codes, demonstration video, and a sample Tag Image File Format (TIFF) image with which to try the program are accessible through links supplied in WorMachine's manual, available Additional file 1.

The *Image Processor* uses still bright-field (BF) images of worm plates as input (acquired using any typical image acquisition microscope). Fluorescent images can similarly be analyzed together with overlapping BF acquisitions. The image acquisition procedures and parameters which enabled optimal processing of images in our hands are detailed in the methods section. Identifying real worms from other elements is normally a painstaking stage that delays image analysis. The Image Processor of WorMachine binarizes, identifies, and crops individual worms from the original image automatically.

The cropped worm masks are then loaded to the *Feature Extractor*, where the worms’ morphological and fluorescent features are analyzed individually. During this stage of the analysis, potentially faulty and damaged worm images are flagged by a deep learning network designed particularly for this task and made available for the investigator’s review. The Feature Extractor also enables tagging different worms with labels according to the user’s needs, such as assigning worms to different conditions or groups. For example, the worms’ sex can be labeled, and this information can be used for creating a training dataset for later classification.

Finally, the *Machine Learner* builds on the obtained features and labels to conduct binary classification, using a support vector machine (SVM), or visualization and scoring of high-dimensional data and dimensionality reduction using principal component analysis (PCA) or *t*-distributed stochastic neighbor embedding (*t*-SNE) [5, 6].

### Image Processor

WorMachine is best suited to handle TIFF images with one or multiple channels, which can have a maximum size of about 1 GB, depending on the memory of the user’s computer. However, it also supports a wide range of additional formats used by biologists, as it incorporates the Bio-Formats Library [7] for image reading. Imported images are automatically grayscaled and contrast-adjusted to accentuate the differences between worms and the background and accordingly to improve the detection of nematodes. A representative input TIFF image with multiple worms before and after grayscaling is shown in Additional file 2: Figure S1. The program generates a binary mask based on the imported image, using adaptive local thresholding [8], and this mask is then “cleaned” and segmented using MATLAB’s own Image Processing Toolbox (for details, see methods). Individual worms within a likely size are automatically identified, smoothed, filled, and cleaned, using standard image processing procedures included in MATLAB’s Image Processing Toolbox. All individual worm images are numbered and saved to a folder based on their respective image channel (BF, Fluorescence, Masks, etc.). This procedure may be applied automatically on multiple images, using the “Batch Analysis” option (see manual and demo movie link in Additional file 1).

### Feature Extractor

Once individual worm masks are imported into this program, the analysis may be performed on all channels in parallel. All morphological and Fluorescent measurements currently available in WorMachine are detailed in Table 1, and each extraction technique is detailed in the methods section. After extraction, objects which deviate in area size, length, or skeleton disfigurement are flagged for manual inspection, together with images identified as “noise” by the deep learning network. Thus, the user may further clean and refine her database. The proportion of worms excluded by this step is detailed in Additional file 3: Table S1.

**Table 1:** Morphological and fluorescent features and their calculation methods

Feature	Calculation
Area	Number of pixels in the worm's area × pixel height × pixel width
Length	Number of pixels in the worm's skeleton × pixel height
Thickness	Area/length
Midwidth	Number of pixels in the worm's center width × pixel height
Head and tail diameter ratios	$\frac{\mathrm{Diameter}\;\mathrm{at}\;\mathrm{thickest}\kern0.17em \mathrm{section}\;10\%\mathrm{from}\kern0.17em \mathrm{edge}}{\mathrm{Diameter}\;\mathrm{at}\;\mathrm{slimmest}\kern0.17em \mathrm{section}\;10-20\%\mathrm{from}\kern0.17em \mathrm{edge}}$
Head Bright-Field	Mean brightness of a polygon within the worm's head
Tail Bright-Field	Mean brightness of a polygon within the worm's tail
Peaks count	2d local maximum of fluoresecent intensities
Mean and STD of peaks	Average and standard deviation of peak intensities
Corrected Total Worm Fluoresence (CTWF)	Mean worm fluorescence × worm area – mean background fluorescence × worm area
Raw Integrated Density (RID)	Sum of worm fluorescence in its area – mean background fluorescence × worm area

### Machine Learner

At this stage, data extracted from the previous stages can be analyzed with different Machine Learner techniques. First, users may visually review and select features relevant to their analysis. Next, the user can choose between two techniques: (1) SVM for binary classification based on supplied or user-generated training data or (2) high-dimensionality visualization and scoring of complex phenotypes based on various features using PCA or *t*-SNE. The algorithms and their use are detailed in the methods section, and examples for different applications are provided in the Results section.

## Results

We examined WorMachine’s ability to facilitate analyses of multiple different phenotypes. First, we describe the use of the software for classification of worm populations based on binary-sex phenotypes (males or hermaphrodites). Then we demonstrate, using a mutant that displays a continuous-sexual phenotype, that WorMachine can accurately create a common scale of worm masculinization. Next, we show how the software can be used to quantify RNA interference (RNAi)-induced gene silencing, protein aggregation, and puncta distribution.

### Binary classification of the worms’ sex

**Fig. 2:**
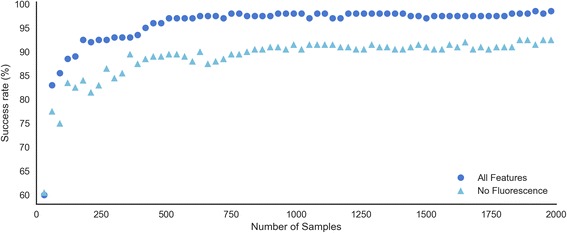
Success rates in classifying the worms’ sex as a function of the number of worms used for training. Shown are results when the fluorescence of a reporter expressed in sex-specific neurons was taken into account (*dark blue, dots*) and when only morphological and brightness features were considered (*light blue, triangles*). The classification is based on these morphological features: head BF, tail BF, area, length, midwidth, thickness, tail ratio, and on the following fluorescent features: peak number (count), corrected total worm fluorescence (CTWF), and mean peak intensity. The training set included 30–2000 worms, while the test set included 200 worms, 100 of each sex, randomly selected and excluded in advance

*C. elegans* nematodes have two sexes - the majority of the worms are self-fertilizing XX hermaphrodites, and a small minority (0.1–0.2%) are X0 males [9]. WorMachine can be used to calculate in a high-throughput and precise manner the sex ratios in different strains and conditions. To distinguish between the sexes, WorMachine uses morphological and brightness features that differentiate between hermaphrodites and males and, also, when fluorescent reporters are available, sex-specific expression patterns. The mutant worms that we used here (*him-5(e1467)*; *zdIs13 [tph-1p::GFP] IV])* segregate many males and express green fluorescent protein (GFP) in the serotonergic neurons. Mutations in *him-5* increase the frequency of XO males (to about 30%) by elevating the frequency of X chromosome nondisjunction [10]. The *tph1p::GFP* transgene allows one to distinguish the worms’ sex as it drives GFP expression in male-specific and hermaphrodite-specific neurons: the hermaphrodite-specific neuron (HSN), the males’ ventral cord motor neurons (CPs), and some tail-specific neurons [11, 12]. We classified worms based on morphological, brightness, and fluorescent features (Additional file 4: Figure S2) and reached 98% classification accuracy when we trained on all features using 1800 worms. Figure 2 displays the true positive rates of the machine learning program, based on training sets of different sizes (30 to 2000 worms), with and without taking advantage of the sex-specific Fluorescent pattern. For each size of the training set on which the model was trained, the true positive rates presented were based on predictions the model performed on the same held-out test set; the test set was composed of 200 worms, 100 males and 100 hermaphrodites, randomly selected and excluded from the entire dataset in advance of any training.

### Quantifying a continuous-sex phenotype

**Fig. 3:**
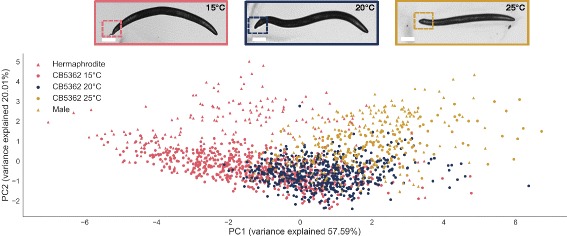
A PCA visualizing the effect of temperature on the sexual phenotype. CB5362 worms were grown in different temperatures and imaged during the first day of adulthood. The PCA was calculated on scaled data and was based on the statistically significant and theoretically justified features that distinguish between the sexes (e.g., area, length, midwidth, thickness, tail ratio, head BF, and tail BF, as shown in Additional file 2: Figure S1A). *Circular data points* represent individual worms of the CB5363 strain displaying a temperature-dependent intersex phenotype. *Triangular data points* represent individual worms with binary-sex phenotype (BFF23). *Red circles* = 15 °C; *blue circles* = 20 °C; *yellow circles* = 25 °C. Triangles representing binary-sex phenotype worms: *red triangles* = males, *yellow triangles* = hermaphrodites. *Upper panel* contains representative worms at each temperature. Scale bar = 100 μm

Continuous morphological phenotypes are common, and due to their complex nature, their quantification is often challenging. We used the CB5362 strain, which is mutated in the sex determination genes *xol-1* and *tra-2*. These worms display an intersex phenotype which depends on temperature [13]. We used WorMachine to determine the sexual phenotype (= degree of masculinization) of each worm, based on multiple features: the shape of the tail (angle evaluation) [14], the presence or absence of eggs in the gonad (egg-bearing worms have larger midwidth), the worm's length and area (males are smaller than hermaphrodites), and the head and tail brightness (males have darker tails in BF) (Additional file 4: Figure S2). We grew CB5362 worms at three different temperatures (15, 20, and 25 °C) and imaged them at the first day of adulthood. The program determined the degree of masculinization of each worm, ranging from male to hermaphrodite, using dimensionality reduction techniques. PCA yielded scores that were concurrent with previous literature, showing higher masculinity scores for higher temperatures (Fig. 3, Additional file 5: Figure S3). *t*-SNE analysis yielded similar results (Additional file 6: Figure S4). In order to verify that the spread of the first PCA component, as illustrated in Fig. 3, is indeed a measure of masculinization degree, we added worms with binary-sex phenotype to the PCA. The figure shows that the higher the temperature in which the continuous-sex phenotype worms were grown, the more they resemble males from the binary-sex phenotype strain in the first principal component.

### Quantifying RNAi-induced phenotypes

We used WorMachine to quantify the RNAi response of worms fed with anti-*dpy-11* or anti-*mCherry* double-stranded RNA (dsRNA) expressing bacteria.

#### Anti-dpy-11 RNAi

**Fig. 4:**
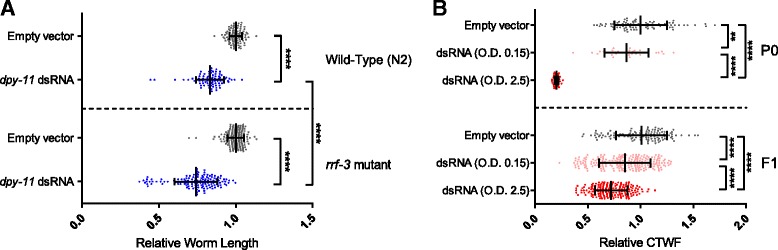
Quantification of RNAi-induced phenotypes. The worms were imaged during the first day of adulthood. The values for each worm were divided by the mean value of the corresponding control group. Each *dot* represents an individual worm. *Bars* represent mean ± standard deviation (****p < 10^–4^, **p < 10^–2^, one-way analysis of variance (ANOVA) test with Bonferroni post hoc correction). **a** Quantification of the Dumpy phenotype following *dpy-11* RNAi treatment. N2 worms (*upper panel*) or *rrf-3* mutants (*lower panel*) were fed with bacteria expressing an empty vector control or dsRNA complementary to *dpy-11*. **b** Quantification of fluorescence intensity in whole animals. EG7841 worms expressing mCherry in all somatic cells were fed with bacteria expressing either an empty vector control or dsRNA complementary to mCherry. The RNAi-producing bacteria were grown to the indicated optical density (*O.D.*). P0 condition: The eggs were laid on RNAi-producing bacteria lawns. F1 condition: The progeny of the RNAi-treated worms that were laid and grown on standard OP50 bacteria

Knockdown of *dpy-11* results in a “Dumpy” phenotype (reduced length) [15]. In addition to using wild-type worms (N2), we also examined the RNAi response in *rrf-3(pk1426)* mutants, which are hypersensitive to RNAi (they exhibit an enhanced RNAi, or Eri, phenotype) [16]. As can be seen in Fig. 4a, WorMachine successfully captures the stronger response to RNAi of *rrf-3* mutants, in comparison to N2 wild types (*p* < 10^–4^).

#### Anti-mCherry RNAi

We used worms that express mCherry ubiquitously (EG7841 *oxTi302 [eft-3p::mCherry::tbb-2 3'UTR + Cbr-unc-119(+)]* [17]. Worms were treated with dsRNA-expressing bacteria grown to different optical density (OD) values (to obtain a gradient of silencing efficiencies), and the corrected total worm fluorescence (CTWF) was measured. As expected, worms exposed to bacteria grown to higher OD values showed lower CTWF values which reflect greater levels of silencing (*p* < 10^–4^) (Fig. 4b). The differences in CTWF values (silencing levels) that were automatically measured by WorMachine were comparable to the differences measured when using ImageJ on manually defined worms (Additional file 7: Figure S5).

### Quantification of intracellular protein aggregation

**Fig. 5:**
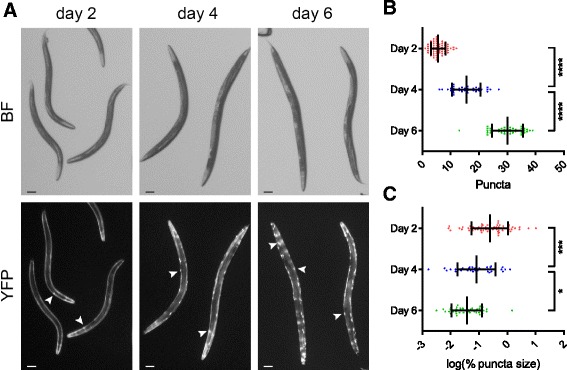
Quantifying intracellular fluorescent protein aggregation. **a** Representative images of polyQ35-YFP-expressing animals at days 2, 4, and 6 of adulthood. *BF* bright-field illumination. Fluorescent protein aggregations are marked with *white arrows*. Scale bars = 100 μm. **b** Quantification of polyQ35-YFP puncta at days 2 (*red*, *n* = 64), 4 (*blue*, *n* = 37), and 6 (*green*, *n* = 42) of adulthood. Each *dot* represents an individual worm. *Bars* represent mean ± standard deviation (****p < 10^–4^, one-way ANOVA test with Bonferroni post hoc correction). **c** Quantification of the relative sizes of polyQ35-YFP puncta on days 2 (*red*), 4 (*blue*), and 6 (*green*) of adulthood. Values for mean puncta size per worm as percent of worm size were log (base e) transformed. Each *dot* represents an individual worm. *Bars* represent mean ± standard deviation (***p < 0.001, *p < 0.05, one-way ANOVA test with Bonferroni post hoc correction)

Protein aggregation can be toxic and is a hallmark of many diseases [18]. The Cohen lab (at the Hebrew University of Jerusalem) studies the cellular mechanisms of polyglutamine toxicity and agreed to test whether WorMachine can be useful for quantifying the aggregation of polyglutamine proteins. Importantly, the analysis of this phenotype and the data acquisition were done outside of the Rechavi lab, using a different microscope, and by non-Rechavi lab members (Amir Levine, from the Cohen lab). The transgenic AM140 worm strain expresses a polyglutamine protein (35 repeats) tagged with the yellow fluorescent protein (polyQ35-YFP) in body wall muscles [19]. These animals form visible polyglutamine puncta that accumulate in an age-dependent manner. The sizes and quantity of these puncta serve as a measure for toxic polyglutamine aggregation [19, 20]. The large variability of puncta quantities among worms in a population and the large differences in puncta sizes within each individual worm normally require the collection of large datasets to achieve reproducible and consistent results. WorMachine was able to measure the number and size distributions of polyQ35-YFP in a high-throughput manner. The abundance of polyQ35-YFP puncta increases with age (in accordance with the literature), while the relative sizes of polyQ35-YFP puncta decrease (Fig. 5). The differences in the number of puncta between the different experimental conditions (different days) that were identified manually were also identified by WorMachine (Additional file 7: Figure S5).

## Discussion and conclusions

WorMachine offers the nematode research community an easy-to-use, automated, accurate, and reproducible methodology to analyze morphological and fluorescent worm features from images obtained using standard microscopes. The software is free and has a modular design in each step that is adaptable to user-specific requirements.

Other useful software programs for analysis of *C. elegans* images exist. The most similar existing software is WormToolbox [4], a module created for CellProfiler [21], which also enables high-throughput phenotypic analysis of morphology and fluorescence. In contrast to WormToolbox, WorMachine is entirely MATLAB-based, and it was built to provide a quick and easy alternative, with an interface that less computer literate users can appreciate. In addition, WormToolbox is adjusted for images of worms suspended in liquid (for example, in 96-well plates) and handles lower quantities of worms per image (up to 15 worms per well). Although WormToolbox is very useful in some contexts, it may be helpful for researchers to use WorMachine, which captures large quantities of worms in 60-mm agarose plates and enables extensive batch processing. In fact, our software is only limited by the image file size (depending on the user’s computer memory capacity). Importantly, WorMachine introduces the first, to our knowledge, neural network that is trained to distinguish worms from noise. The deep learning neural network implemented in WorMachine recognizes, flags, and omits non-suitable objects, to ensure a high level of quality for the users’ generated data.

Further advantages WorMachine offers are the machine learning algorithms provided in its Machine Learner section, which can uncover new information about the data that would be hard to reveal using standard analysis. For example, scoring the continuous-sex phenotype based on the program’s output features provides information on degree of masculinity that is unattainable through each feature on its own. More generally, the dimensionality reduction techniques allow clear visualization of any dataset obtained through WorMachine for easier interpretation.

There are many more possible applications for WorMachine in addition to quantification of the biological features that we analyzed in this paper. Firstly, the variety of details the software affords enables researchers to easily examine novel relations between them, such as the association between fluorescent markers to morphological features, and determine the most appropriate and artifact-free measure. In addition, the modular design of the software allows adaptation of its algorithms on many experimental datasets. For example, SVM models can be applied on any binary phenotype or experimental manipulation to automatically classify large sets of worms out of a customized limited training dataset. As for complex continuous phenotypes, such as developmental stages or various fluorescent patterns, PCA or *t*-SNE can be applied on custom datasets to obtain an aggregate continuous score. We hope that many users will find this software useful in the near future.

## Methods

### Preparation of worms for imaging

Worms were synchronized at each generation in one of two standard ways: (1) mothers were allowed to lay eggs for a limited span of 24 h, or (2) bleaching (“egg-prep”) was performed [22]. Adult worms were washed three times to get eliminate bacterial (OP50) residues. Worms were left in ~ 100 μL of M9 buffer and paralyzed via the addition of sodium azide (final concentration of 25–50 mM). The paralyzed worms were transferred to imaging plates and then physically separated from each other using a platinum-wire pick. Separation of the worms is important for allowing the software to correctly process each worm individually. The imaging plates were 60-mm petri dishes filled with 8 mL of modified transparent nematode growth medium (NGM, 2% agarose, 0.3% NaCl, 5 mM K_2_PO_4_, 1 mM CaCl_2_, 1 mM MgSO_4_).

### Microscopy image acquisition

Images were taken on an Olympus IX83 microscope, using fluorescence excitation with a light-emitting diode (LED) light source on two channels: GFP and mCherry fluorescence. For BF imaging, a relatively long exposure time was used for trail erasure, and the contrast was increased to better differentiate between worms and background. Pictures were taken with a 4X/0.75 Universal Plan Super Apochromat objective.

For the protein aggregation assays, images were acquired using a Nikon SMZ18 stereoscope fitted with a 1X objective, set up to capture both BF illumination and YFP fluorescence.

### Measurement of worm length following *dpy-11* RNAi treatment

For this assay, we took images of live worms on NGM plates. The worms grew on the indicated treatment after synchronization by egg laying. At the first day of adulthood, the worms went through four rounds of washes in M9 buffer and were transferred to a new NGM plate. Images of worms were obtained using a DCM-310 digital camera (Scopetek) attached to an SMZ745 stereomicroscope (Nikon) with its objective set to 2X magnification. The ScopePhoto software was used for image acquisition. Images were then loaded to WorMachine’s Image Processor using the available preset global setting for low-resolution images. Further analytical steps were performed similarly as in all other presented assays.

### *C. elegans* strains

The *C. elegans* strains employed in this work are as follows: wild-type Bristol N2 strain, BFF23: *him-5(e1490)* V; *zdIs13(tph-1p::GFP)* IV; CB5362: *tra-2(ar221)* II; *xol-1(y9)* X, AM140: *rmIs132(unc-54p::Q35-YFP)* I; NL2099: *rrf-3(pk1426)*; EG7841: *oxTi302 [eft-3p::mCherry::tbb-2* 3'UTR + Cbr-*unc-119(+)]*.

### RNAi treatment

We used a standard RNA interference (RNAi) feeding protocol, as previously described [22]. In each stage of the different experiments, worms were cultivated either on HT115 bacteria that transcribe a specific dsRNA (e.g., targeting *mCherry* or *dpy-11*) or on control HT115 bacteria that contain only an “empty vector” that does not lead to dsRNA transcription. The NGM plates contained isopropyl ß-d-1-thiogalactopyranoside (IPTG) for induction of dsRNA expression. The offspring which hatched on these plates were examined.

### Image requirements and limitations

WorMachine was tested on a variety of image resolutions, signal-to-noise ratios, bit depths, and contrasts. The software offers three preset global settings, which set all necessary image processing parameters according to a given microscope magnification used to acquire the image: high ($10\mathrm{X}\;\mathrm{Objective}-0.45\frac{\mu m}{pixel}$), medium ($4\mathrm{X}\;\mathrm{Objective}-1.14\frac{\mu m}{pixel}$), and low ($2\mathrm{X}-5.14\frac{\mu m}{pixel}$). The software has been successful with resolutions of 96, 72, and up to very small dpi, and bit depths of 8, 16, and 24. However, as internal computer memory is limited, loading high-resolution images, which are usually very exhaustive in memory uptake, can be problematic. Thus, regardless of resolution, bit depth, and contrast, the software is limited to working on ~ 1 GB per image. Further testing showed that worms were successfully processed and analyzed in almost any posture, including omega turns (see examples in Additional file 8: Figure S6). However, worms that complete a full circle, such that one of their ends touches some point on their body, cannot be analyzed with our software and will be flagged as faulty. Worms that touch another worm or object directly would also be excluded, so the best practice is to separate touching worms on the plate before acquiring images. Also, we found that using plates with agarose instead of agar provided images with better foreground-background separation, since it allows more light to pass through the plate’s surface where no worms are present, increasing the background’s brightness relative to the worms on the plate. Lastly, using images with contrast lower than 0.04 (defined as a grayscaled image’s standard deviation [23]) is not possible, and therefore worms should be imaged on plates rather than slides (which typically have lower contrast). The software successfully analyzes images of plates with higher contrast, ranging from 0.06 to 0.15; increasing the contrast further usually provides better results, as long as the image is not distorted by amplified noise.

### Image binarization, cleaning, and segmentation

A mask image is produced by applying adaptive local thresholding [8], using the free parameters ‘neighborhood’ and ‘threshold’. The first determines the number of pixels around a given pixel that would be considered when deciding its value, while the second sets the relative intensity for thresholding the given pixel. The mask image is a matrix of equal size as the original image, containing ones where a suspected worm is present and zeros in the background. The filter’s parameters ‘neighbors’ and ‘threshold’ may be adjusted manually, though several default recommended presets are offered, per the image’s resolution. After binarization, objects with areas smaller than a specified value will be deleted, including objects touching the edges of the image, which are unlikely to be whole worms. All the objects in an image are identified using MATLAB’s “regionprops” function and are considered worms only if their area is within a specified relative range.

### Flagging faulty worms

#### Morphological outliers

Aberrant worms are flagged using two methods. First, the program locates outliers — worms with extremely large or small areas or lengths or with non-continuous skeletons. All worms’ lengths and areas are standardized, and those that deviate by more than 1.5 or less than –1.5 standard deviation units from the mean are flagged. Moreover, when the worm’s skeleton is extracted, the worm is smoothed using a median filter until a singular continuous skeleton line is obtained, for a maximum of five attempts. If more than two attempts were required to obtain a smooth skeleton from a worm mask image, or, if after the maximal smoothing attempts the skeleton still contains branch points, the mask image is likely to be disfigured or aberrant and will be flagged.

#### WormNet

**Fig. 6:**
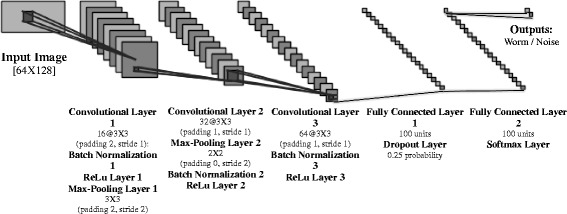
WormNet architecture. The network receives an input image of size 64 × 128 pixels and outputs a classification into worm or non-worm (noise) categories. The complete architecture is detailed in the figure and can also be viewed within the WorMachine’s open-source code. WormNet was trained on 11,820 mask images of worms and non-worms, randomly split into 85% training and 15% test set. It classified with 99.2% accuracy on the training set and 93.4% on the held-out test set

A dedicated *convolutional neural network (C-NN)* was developed particularly for this task. This network has been trained on 11,820 mask images containing equal amounts of “valid” worms and noisy objects or worms with a variety of faulty features. The images were collected from various experiments in our lab in which WorMachine was used for their analysis. Experimenters manually reviewed their worm images, and they discarded images that were defective for different reasons. The discarded images were labeled as faulty, and the remaining images as valid. Mask images were first padded to the size of the 65th percentile mask size, and all were rescaled to the network’s defined input size of 64 × 128 pixels. Worms were then split to 85% training set and 15% hold-out test set. Training was performed with the stochastic gradient descent with momentum optimizer, 0.9 momentum, an L2 regularization of 0.0001, a mini-batch size of 256, and a learning rate of 0.005, for 50 epochs while data was shuffled every epoch. This yielded 99.2% training accuracy and 93.4% accuracy on the test set. The network’s complete architecture is depicted in Fig. 6. Thus, any user-generated mask images may be automatically padded and rescaled in the same procedure and then classified into “worm” or “non-worm” categories, clearly marked in the program’s interface for the users’ convenience. Moreover, the program allows users to retrain the network on their own labeled data, to further improve and fit the network to their laboratories’ specifications.

### Morphological measures

The calculation of the worms’ morphological features includes several steps. First, the worm is skeletonized to a single line using the “bwmorph” function and “thin” argument; the line is then cleaned and pruned of branches to obtain the worms’ continuous skeleton line. The debranching procedure is performed by applying a [15 15] median filter on a temporary copy of a mask image, if any branch points are found on the worm’s skeleton, followed by another “thin” command. If branch points are still found, the temporary mask is smoothed and thinned again, until all branch points are gone or until five attempts were made. Afterwards, the worms’ edges are located using the “edge” function with the Sobel technique. A worm’s area is calculated using the “bwarea” function on the mask image, which is then multiplied by pixel height and pixel width. Its length is calculated by using the same function on the skeleton, which is then multiplied by pixel width. A pixel’s height and width are given as editable input in the program’s interface. Thickness is calculated by dividing the area by the length. Middle-width (Midwidth) is computed using our own “cross_section” function, which locates the length of the worm’s cross section which is perpendicular to the pixel in the exact center of the skeleton. Lastly, the extraction of head and tail diameter ratios was adapted from WormGender [24], but with some modifications. The software calculates the mean intensity in the BF image around the two ends of the worm’s skeleton. It was previously shown [25] that ends with higher intensity are characteristic to the head of the worm. Tests we performed on pre-labeled heads and tails of 500 worms showed this method to be accurate in 85% of worms. In order to further improve robustness of head-tail distinction, we took advantage of a known morphologically distinct phenotype. Male worms have tails with larger diameter than heads, while hermaphrodites have tails with smaller diameter than heads [24]. We combined this information with head and tail brightness and achieved 95% accuracy of distinction between heads and tails in our testing. The first diameter of each end (D1) is the *longest* cross section found 10 pixels from the worm’s end and up to 10% of the worm’s length. If the longest cross section is at 10% of its length, then the length of the cross section at 2.5% of the worm’s length is taken as D1. The search for the second diameter of each end (D2) begins 20 pixels from D1’s location and continues up to 20% of the worm’s length, until the *shortest* cross section is found. Lastly, the diameter ratio for each end is calculated by dividing D1 by D2. We developed this algorithm to maximize the diameter ratio of the wider male tails [25], without biasing against hermaphrodites’ tails, to improve distinctiveness between sex phenotypes. Following these adjustments, our software distinguishes the worms’ sex successfully in 98% of the cases tested (see the Results section for more details).

### Fluorescent analysis

We applied MATLAB’s “LocalMaximaFinder” object (part of the “Computer Vision” package) to locate the local maximum intensities (peaks) throughout the image, using the adjustable parameters Neighborhood and Threshold. The Neighborhood parameter specifies the size of a square surrounding an identified peak, in which no other local maxima can be considered as peaks. A large Neighborhood allows only peaks that are brightest and furthest apart to be identified, while a small neighborhood allows many adjacent peaks to be identified separately. The Threshold parameter enables control over the minimal intensity that can be considered as a peak, and it is set by choosing the desired percentage from the image’s maximum intensity. The number of peaks identified, their mean intensity, and standard deviation are reported for each worm. In addition, the raw integrated density (RID) is calculated by summing the intensity values of all pixels within the worm’s area and subtracting the mean background intensity multiplied by the number of pixels in the worm’s area. Lastly, the CTWF is calculated by subtracting the mean intensity within the worm from the mean intensity of its background, which is then multiplied by the worm’s area.

### Binary classification

The creation of a SVM model for binary classification is available, based on a labeled dataset generated by the Feature Extractor. Users may choose a kernel method, whether or not to standardize their data, and the number of cross validations on the data, aimed at reducing model overfitting. The program splits the data into a training set and a test set and performs optimization towards an appropriate SVM model. The resulting model may be later used to classify unlabeled datasets with similar features into the labels on which it was trained. One may create one’s training dataset by manually labeling worms and then use the model created based on the data to classify unlabeled worms. Alternatively, one may utilize the dataset to obtain prediction rates for various combinations of features, in order to assess the contribution of each feature towards accurate prediction (Fig. 2). We supply a trained model for the purpose of worm sex classification, but we recommend customized labeling and training to create bespoke models that would better suit each lab’s specifications.

### Unsupervised scoring

The technique called *t*-SNE [5], for dimensionality reduction, is particularly well suited for the visualization of high-dimensional datasets and gives each data point a location on a two- or three-dimensional map. The technique is a variation of stochastic neighbor embedding that is easier to optimize and produces better visualizations [26]. This method of unsupervised learning essentially enables scoring data within a single common dimension, giving each sample a continuous value. The data is pre-processed using PCA [6], reducing the dimensionality. Later, the dimensionality is reduced again via the *t*-SNE technique. If the data is indeed labeled, although labels are not used by *t*-SNE itself, the labels can be used to color the resulting plot. The algorithm’s final output is the low-dimensional data representation.

## Additional files

Additional file 1: WorMachine Manual, including links for Demo Video, Software Codes, and Sample TIFF Image. (PDF 737 kb)

Additional file 2: Figure S1. Typical input image. A standard image as acquired by the microscope (A) and after grayscaling (B). Scale bar 1 mm. Both images were compressed for their addition to the paper, but their original size was 1.1 GB. The resolution was 72 dpi with 16 bit depth, and the dimension was 17,610 × 16,116. (PPTX 355 kb)

Additional file 3: Table S1. Reliability of worm identification. Results showed percentage of worm retention from initial object count. This demonstrates that once objects are identified by the Image Processor, they are likely to be identified as a “real worm” rather than be removed as noise. (DOCX 11 kb)

Additional file 4: Figure S2. Features used to establish worm masculinity. Violin plots show the morphological features (A) and fluorescent features (B) used to determine the masculinity of *him-5*; *[tph-1p::GFP]* worms. A total of 545 pre-labeled worms of each sex were used for analysis (****p < 10^–4^, ***p < 10^–3^, *p < 0.05, two-tailed *t* test after α = 0.01 trimming to exclude extreme outliers with false discovery rate (FDR) correction for multiple comparisons). Only features that were significantly distinct and had plausible theoretical justification to differentiate between sexes were used for sex phenotype prediction. As can be seen in this figure, males and hermaphrodites differ in some features but not in every feature examined. (PPTX 597 kb)

Additional file 5: Figure S3. Features contribution. Demonstrates the relative contribution of each feature to the separation across the first component of the PCA in Fig. 3. (PNG 13 kb)

Additional file 6: Figure S4. *t*-SNE visualizing the effect of temperature on the sexual phenotype. The same assay presented in Fig. 3 analyzed with *t*-SNE instead of PCA. (PNG 692 kb)

Additional file 7: Figure S5. Comparisons between scoring of different phenotypes using WorMachine and alternative scoring methods. A. CTWF measurements, taken either manually using ImageJ or automatically by WorMachine. B. Number of puncta, counted manually, or automatically by WorMachine. (PPTX 296 kb)

Additional file 8: Figure S6. Various worm images demonstrating WorMachine’s capabilities and limitations. A. Images show the software’s ability to skeletonize and analyze worms in a variety of postures. B. The software is limited in identifying worms that touch another worm or any point on their own body. These are flagged as faulty. Scale bar indicates 50 μm. (PPTX 204 kb)

## References

[1] Moore BT, Jordan JM, Baugh LR, Byerly L, Cassada R, Russel R (2013). WormSizer: high-throughput analysis of nematode size and shape. PLoS One.

[2] Schindelin J, Arganda-Carreras I, Frise E, Kaynig V, Longair M, Pietzsch T (2012). Fiji: an open-source platform for biological-image analysis. Nat Methods.

[3] Jung S-K, Aleman-Meza B, Riepe C, Zhong W, Mathew M, Mathew N (2014). QuantWorm: a comprehensive software package for Caenorhabditis elegans phenotypic assays. PLoS One.

[4] Wählby C, Kamentsky L, Liu ZH, Riklin-Raviv T, Conery AL, O’Rourke EJ (2012). An image analysis toolbox for high-throughput C. elegans assays. Nat Methods.

[5] van der Maaten L, Hinton G (2008). Visualizing data using t-SNE. J Mach Learn Res.

[6] Jolliffe I (2014). Principal component analysis.

[7] Linkert M, Rueden CT, Allan C, Burel J-M, Moore W, Patterson A (2010). Metadata matters: access to image data in the real world. J Cell Biol.

[8] Bradley D, Roth G (2007). Adaptive thresholding using the integral image. J Graph Tools.

[9] Corsi AK, Wightman B, Chalfie M (2015). A transparent window into biology: a primer on Caenorhabditis elegans. Genetics.

[10] Hodgkin J, Horvitz HR, Brenner S (1979). Nondisjunction mutants of the nematode Caenorhabditis elegans. Genetics.

[11] Shyn SI, Kerr R, Schafer WR (2003). Serotonin and Go modulate functional states of neurons and muscles controlling C. elegans egg-laying behavior. Curr Biol.

[12] Loer CM, Kenyon CJ (1993). Serotonin-deficient mutants and male mating behavior in the nematode Caenorhabditis elegans. J Neurosci.

[13] Hodgkin J (2002). Exploring the envelope: systematic alteration in the sex-determination system of the nematode Caenorhabditis elegans. Genetics.

[14] Chandler CH, Phillips PC, Janzen FJ (2009). The evolution of sex-determining mechanisms: lessons from temperature-sensitive mutations in sex determination genes in Caenorhabditis elegans. J Evol Biol.

[15] Ko FCF, Chow KL (2002). A novel thioredoxin-like protein encoded by the C. elegans dpy-11 gene is required for body and sensory organ morphogenesis. Development.

[16] Simmer F, Tijsterman M, Parrish S, Koushika SP, Nonet ML, Fire A, Ahringer J, Plasterk RH (2002). Loss of the putative RNA-directed RNA polymerase RRF-3 makes C. elegans hypersensitive to RNAi. Current biology.

[17] Frøkjær-Jensen C, Davis MW, Ailion M, Jorgensen EM (2012). Improved Mos1-mediated transgenesis in C. elegans. Nat Methods.

[18] Kopito RR (2000). Aggresomes, inclusion bodies and protein aggregation. Trends Cell Biol.

[19] Morley JF, Brignull HR, Weyers JJ, Morimoto RI (2002). The threshold for polyglutamine-expansion protein aggregation and cellular toxicity is dynamic and influenced by aging in Caenorhabditis elegans. Proc Natl Acad Sci.

[20] Caldwell GA, Cao S, Sexton EG, Gelwix CC, Bevel JP, Caldwell KA (2003). Suppression of polyglutamine-induced protein aggregation in Caenorhabditis elegans by torsin proteins. Hum Mol Genet.

[21] Carpenter AE, Jones TR, Lamprecht MR, Clarke C, Kang I, Friman O (2006). Cell Profiler: image analysis software for identifying and quantifying cell phenotypes. Genome Biol.

[22] Stiernagle T. Maintenance of C. elegans. WormBook. 2006. http://www.wormbook.org/chapters/www_strainmaintain/strainmaintain.html. Accessed 23 Oct 2017. 10.1895/wormbook.1.101.1 PMC4781397 18050451

[23] Moulden B, Kingdom F, Gatley LF (1990). The standard deviation of luminance as a metric for contrast in random-dot images. Perception.

[24] Labocha MK, Jung S-K, Aleman-Meza B, Liu Z, Zhong W (2015). WormGender — open-source software for automatic Caenorhabditis elegans sex ratio measurement. PLoS One.

[25] Geng W, Cosman P, Huang C. Automated worm tracking and classification. 2003. Asilomar Conference on Signals, Systems and Computers, IEEE, 2004. http://citeseerx.ist.psu.edu/viewdoc/download;jsessionid=3B3800028E12BA1492A95642DE70A3EB?doi=10.1.1.659.9101&rep=rep1&type=pdf. Accessed 23 Oct 2017.

[26] Hinton GE, Roweis ST. Stochastic neighbor embedding. Advances in Neural Information Processing Systems 15 (NIPS 2002). p. 833–40. http://papers.nips.cc/paper/2276-stochastic-neighbor-embedding.pdf.

