# Supplementary material for: WorMachine: machine learning-based phenotypic analysis tool for worms
Source: BMC Biol. 2018 Jan 16;16:8. doi: 10.1186/s12915-017-0477-0 (PMC5769209; doi:10.1186/s12915-017-0477-0)
Supplement: Supplementary file 1 — WorMachine Manual, including links for Demo Video, Software Codes, and Sample TIFF Image. (PDF 737 kb) [file 12915_2017_477_MOESM1_ESM.pdf]

WORMACHINE

MACHINE LEARNING-BASED PHENOTYPIC ANALYSIS TOOL FOR WORMS

MANUAL

1. Getting Started 1

1.1 Installation & Links 1

1.2 Startup GUI 1

2. Image Processor 2

2.1 Image Importation & Loading 2

2.2 Auto Batch-Processing 2

2.3 Binarization 3

2.4 Removing Small Objects 3

2.5 Worm Identification 4

2.6 Saving Images 4

3. Feature Extractor 6

3.1 Worms Import 6

3.2 Flagging and Removing Worms 6

3.3 Analyzing Worms 6

3.4 Fluorescent Analysis 7

3.5 Labelling 7

3.6 Exporting WormData 7

3.7 Re-analyze Data 7

4. Machine Learner 9

4.1 Loading & Feature Selection 9

4.2 Classification 9

4.3 Reduction/Scoring 9

4.4 Exporting New Labels 10

1. Getting Started

1.1 – Installation & Links

Installing WorMachine is easy. Simply download all files into a dedicated directory, and run "WorMachine.m" to begin. Make sure not to alter any of the names of the program's scripts, files, folders, GUIs or functions, without appropriate proficiency in MATLAB coding.

Download link:
https://github.com/adamhak/WorMachineClient

View software Demo video:
https://www.youtube.com/watch?v=9HCQ36us-IA

Sample TIFF Image:
https://www.dropbox.com/s/ujhilti4onhbyb1/EG_P0_EV_02.tif?dl=0

Download this image to try out the software with.

1.2 - WorMachine Startup GUI

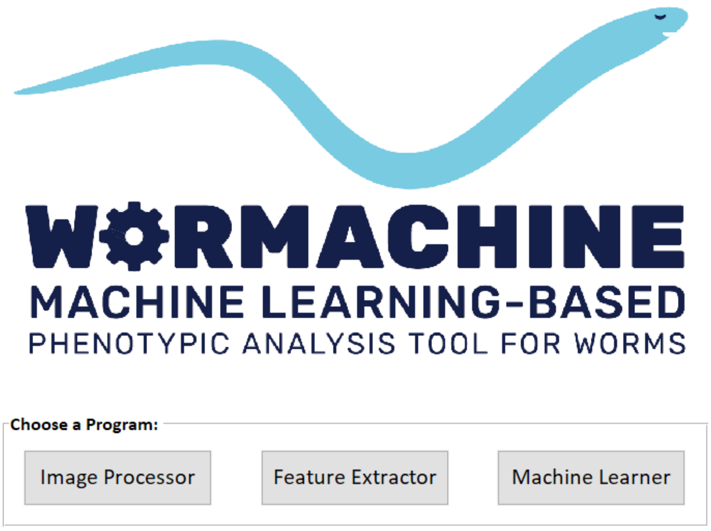

Select the program you wish to use.

2. Image Processor

2.1 - Image Import & Loading

To begin processing an image, first click “Import Image”. Then, choose between:

1. Importing a TIFF file with a single layer/channel – must be a bright-field still image.
2. Importing a TIFF file with multiple layers/channels – a bright-field still image, and any number of overlapping layers/channel (such as fluorescent captures).
3. Importing any Bio-Formats file type.
4. Auto Batch-process multiple TIFF files with multiple layers.

The image’s size should not exceed 1000MB, and should be lower if you do not have enough available RAM. Pressing **Load** will present the image, which is automatically gray-scaled. A list is presented with available channels of the image file loaded. An option to adjust the contrast is available, which may increase the difference in illumination between the foreground (the worms) and background, and thus improve the detection of the worms.

2.2 - Auto Batch-Processing

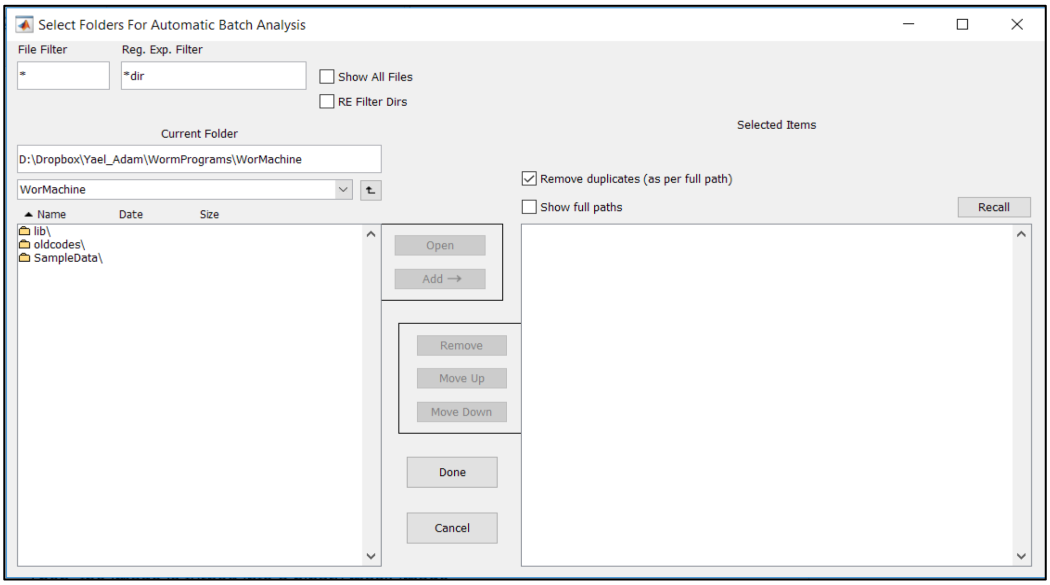

This function is currently available only for TIFF files. Before clicking this option, make sure **all the parameters of the Image Processor program are set** to values suitable to your images, prior to processing. That is, it is recommended to optimize processing parameters for one image first, and then set those parameters for the batch. Once the auto-batch option is pressed, a screen will appear where users may choose which folders contain images for batch-processing. Select these folders in the left window, and press “Add” to move them to the right window, marking them for analysis. Be advised as to how the program handles output on multiple folders and images:

- Each folder is treated separately and will contain its own output. Therefore, pictures representing different experimental groups should be stored in separate folders (but several pictures of one single experimental group may be united in one single folder).
- All images within a certain folder are analyzed together and their outputs are conjoined.
- Worms gathered from different images within a certain folder are saved in the same output folders within that folder. They are numbered continuously from the first worm in the first image of that folder, to the last worm in the last image of that folder.

Once auto-batch analysis starts, steps 2.3 to 2.6 are performed automatically on all chosen images.

2.3 - Binarization

Pressing “Binarize” will generate a binary mask image. The mask image is produced by applying adaptive local thresholding, using the parameters ‘neighborhood’ and ‘threshold’ (explained thoroughly in Bradley and Roth, 2007). The mask image is a matrix of equivalent size as the original image, containing ones where a suspected worm is present, and zeros for the background. The filter’s parameters, ‘neighbors’ and ‘threshold’, may be set manually, though several default recommended presets are offered in the “Preset Settings” tab, per the image’s resolution.

- <u>Neighbors</u> – Determines the number of pixels around a given pixel that would be considered when deciding its binary value. Set as percentage of the image’s width.
- <u>Threshold</u> – Determines the relative intensity for thresholding the given pixel. Set as percentage of the pixel’s intensity.

Low thresholds and low number of neighbors will result in more objects recognized and with larger areas. Higher parameter values will reduce the number of objects and produce smaller and discontinuous worms. Increasing one parameter while reducing the other can result in interchanging effects, possibly producing fuller and finer worms without including too many faulty objects. Thus, it is recommended to change each parameter separately, observe the effect, and optimize them to your image.

2.4 - Removing Small Objects

It is advised to use the ‘Remove Small Object’ button to clean objects with smaller areas than stated by the adjustable parameter “Max Object Areas to Remove”. This parameter is set as the percentage of the image’s area. Objects with area smaller than the given percentage will be turned to zeros. This will also remove objects touching the edges of the image, which are likely to be noise rather than complete worms.

2.5 - Worm Identification

Object are identified using MATLAB's "regionprops" function. Adjustable sliding bars allow to set the range of object areas to be included as worms. By shifting the bars to the right, one may include smaller and larger objects as worms, and by shifting to the left – less.

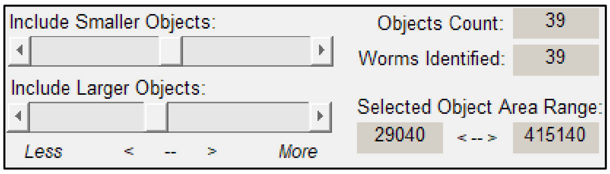

All identified objects are marked with a blue star, yet only objects that have an area size that is within the area range (set by the sliding bars) are also marked with a red box designating them as worms. Objects marked with red boxes will be considered as valid worms, and their bounding box coordinates will be saved for later cropping.

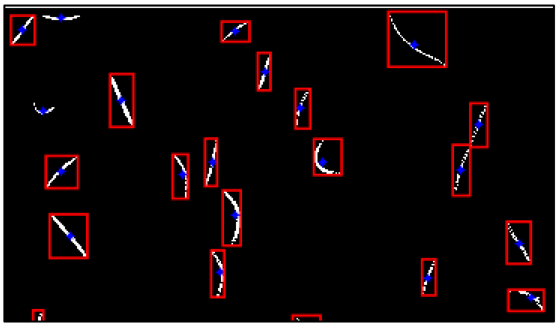

Finally, worms are cropped from their original image, their binary mask image, and any overlapping image that was imported in addition (i.e fluorescent images).

2.6 - Saving Images

Before saving, individual worm masks are automatically smoothed, filled, and cleaned. By ticking the relevant checkboxes, users may choose to save each worm's image cropped from the originally loaded image ('Original'), from the binary image ('Mask') or from any other image channel that overlaps the original image precisely ('Overlap'), such as, for example, a fluorescent image. Masked images are used as input for the calculation of features in the "Feature Extractor". Once "Save Worms!" is pressed, all individual worm images are numbered and saved into a folder that is named with respect to the image channel they originated from ("Originals", "Masks", "Overlap"). Worm images processed and saved into a folder that already contains existing images of worms that were previously processed and saved, will be added to the same output folders on top of the existing images (and will not override them). This is meant to allow the making of a large set of individual worms, derived from various separate images in the same folder, and is used by the software's "Batch Analysis" option. All output folders are saved in the same directory as the original image imported for processing.

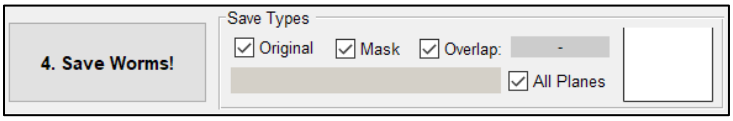

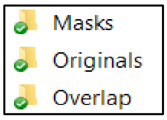

Clicking “N” (Next) on the top-left of the interface, will automatically start the next program, the “Feature Extractor”, already loaded with all processed images.

Since processing a new image produces output images that do not override previous output, be advised that if you plan to process an image again, the former output individual images must be deleted. Otherwise, the new images will be added to the old output, resulting in duplicate outputs.

3. Feature Extractor

3.1 - Worms Import

If worms were not automatically loaded using the “N” button from the Image Processor, then worms must be imported manually. Click “Import” at the top left of the interface, and then press “New Worm Images”. Browse for either your new “Masks” folder, or its parent folder, both will suffice to load all relevant images. To load all worms into the interface, click “Generate Worm Masks”. All worms will appear in a list below the presented mask image. It is possible to scroll through the list to view your worm masks using your mouse or keyboard.

3.2 - Flagging and Removing Worms

Any worm mask image that appears aberrant or faulty may be removed using the “Remove Worm” button or the short-key ‘r’. It is possible to manually flag or unflag worms using the “Flag” button or ‘f’ short-key, and then to remove all flagged worms using the appropriate button. It is also possible to multi-select several worms, using ‘shift’ or ‘ctrl’ keys, and apply these actions on the selected worms. After importing and generating images, pressing “WormNet” will activate the Convolutional Neural Network and classify mask images into valid or faulty worms. In addition, after analysis, the software automatically flags suspicious images using techniques detailed in WorMachine’s paper. All names of flagged worms, as extracted from both methods, are colored yellow. Users may scroll through and view these flagged images only, using the “Next Flagged” button or ‘d’ short-key, in order to unflag or remove them.

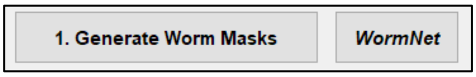

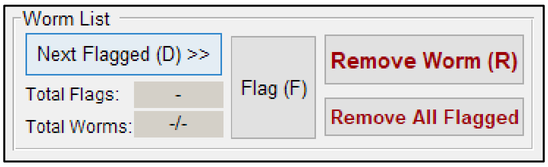

3.3 - Analyzing Worms

Worms are analyzed individually and sequentially for morphological measurements, once the “Analyze Worms” button is pressed. If fluorescent images are also present in the parent folder, they are automatically loaded and analyzed as well, with no additional actions required. Before analysis, it is recommended to input the pixel width and length, as detailed by the acquisition software, in order to correctly transform units from pixels to micrometers. All measurements currently available in our software are detailed in its paper.
Make sure not to touch the program interface while the analysis is running!

3.4 - Fluorescent Analysis

The rightmost panel should present worms' fluorescent images, if present. It is possible to switch to viewing the original image of worms using the short-key '~' or the relevant selection buttons. If fluorescent images were processed but did not load automatically, they may be manually loaded ("Manually load overlapping Images" button) and analyzed ("Analyze fluorescence" button). This analysis is performed using the adjustable parameters – Neighborhood and Threshold. The 'Neighborhood' parameter specifies the number of pixels in the side of a square surrounding a peak, within no other peaks will be allowed to be identified. A large Neighborhood allows only the brightest and most further apart peaks to be considered, while a small neighborhood will allow many adjacent peaks to be identified separately. The 'Threshold' parameter enables control over the minimal intensity that can be considered as a peak, and is set by choosing the desired percentage from the image's maximum intensity.

3.5 - Labelling

After analysis, worms may be labeled as suitable to the users' needs. This may be done on each worm separately, by pressing any number key (0-9) while a worm is selected. It is also possible to label worms with any custom label, by entering the desired label into the appropriate field, and pressing "Set" or the short-key "L". Delete the label by pressing "X' button or shortk-key. Press "Label All" to apply the custom label on all loaded worms at once.

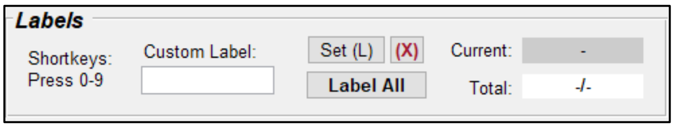

3.6 - Exporting WormData

Users may choose which features they desire to save in the bottom right panel by ticking the relevant checkboxes.

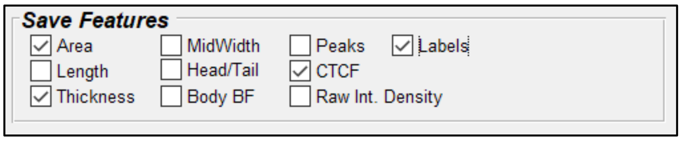

All worm indexes and the selected features may be saved into a MATLAB file, exported to an excel worksheet or a 'csv' file. Choose your preferred option in the "Export" tab at the top left of the interface. All checked features can be saved in 'mat', 'csv' or 'xlsx' formats, such that each column represents a feature, and each row represents an individual worm. It is also possible to add the current data to a previously saved 'mat' file, thus creating a larger dataset within a single file for later analysis. The next program, "Machine Learner", will require input as 'mat' file only. In all exportation options, a column represents a certain feature, while each row represents a different worm.

3.7 - Re-analyze Data

Users may wish to re-analyze a set of images they have already cleaned, labeled and exported beforehand. Thus, after importing the images again, the user may also import the existing dataset (in ‘mat’ or ‘xls’ format) to filter out worm images that were removed in the previous analysis, for the current analysis. In addition, user may choose to extract labels from the existing dataset to apply them on the current analysis.

4. Machine Learner

4.1 - Loading & Feature Selection

Import a worm dataset in ‘mat’ format to begin. Once data is loaded, users may choose whether to standardize all features of the data, and whether to exclude samples which are in the top %5 or bottom 5% in each feature. Features may be viewed using ‘Display Feature’ button, and those desired for further analysis should be moved from the left list to the right list via the “Include” button.

4.2 - Classification

This segment of the program allows the creation of a Support Vector Machine (SVM) model for binary classification, based on a labeled data set generated by the Feature Extractor. Users choose a kernel method and the number of cross validations on the data, aimed at reducing model overfitting. Clicking “Train SVM!” splits the data to a training set and a test set, and performs optimization towards an SVM model of the data. The model may be later used to classify unlabeled datasets with similar features, loaded by pressing “Load New WormData to Classify”. Pressing “Classify” will assign new classes for the loaded dataset, based on the trained data. One may create one’s training dataset by manually labelling samples, and then use the model trained on those samples to classify unlabeled data. Alternatively, one may utilize the data set to obtain prediction rates for various combinations of features, in order to assess the contribution of each feature to the prediction. We supply a trained model for the purpose of sex phenotype classification, but recommend labeling worms and creating a customized model most suited to users’ specifications.

4.3 - Reduction/Scoring

"t-Distributed Stochastic Neighbour Embedding (TSNE) is able to reduce dimensionality and visualize the data. This method of unsupervised learning enables scoring data with a continuous value, as it reduces all data dimensions to a common scale, with a chosen number of dimensions. The data is pre-processed using “Principal Component Analysis” reducing the dimensionality to ‘PCA Dimensions’ (default = 5). Later, dimensionality is reduced again via the t-SNE technique, to the number in ‘t-SNE Dimensions’ (default = 2). The perplexity of the Gaussian kernel that is employed can be specified through ‘Perplexity’ (default = 30). If the data is already labeled, labels are not used by t-SNE itself, however, they can be used to color plots presented in the program.

4.4 - Exporting New Labels

Any new labels created by the program, via any technique, will be saved directly to the ‘mat’ file initially loaded to the software. To do this, simply click “Export” on the top left of the interface, and press “New Labels.
