# Supplementary figures and images for: WorMachine: machine learning-based phenotypic analysis tool for worms
Source: BMC Biol. 2018 Jan 16;16:8. doi: 10.1186/s12915-017-0477-0 (PMC5769209; doi:10.1186/s12915-017-0477-0)

## Slide 1
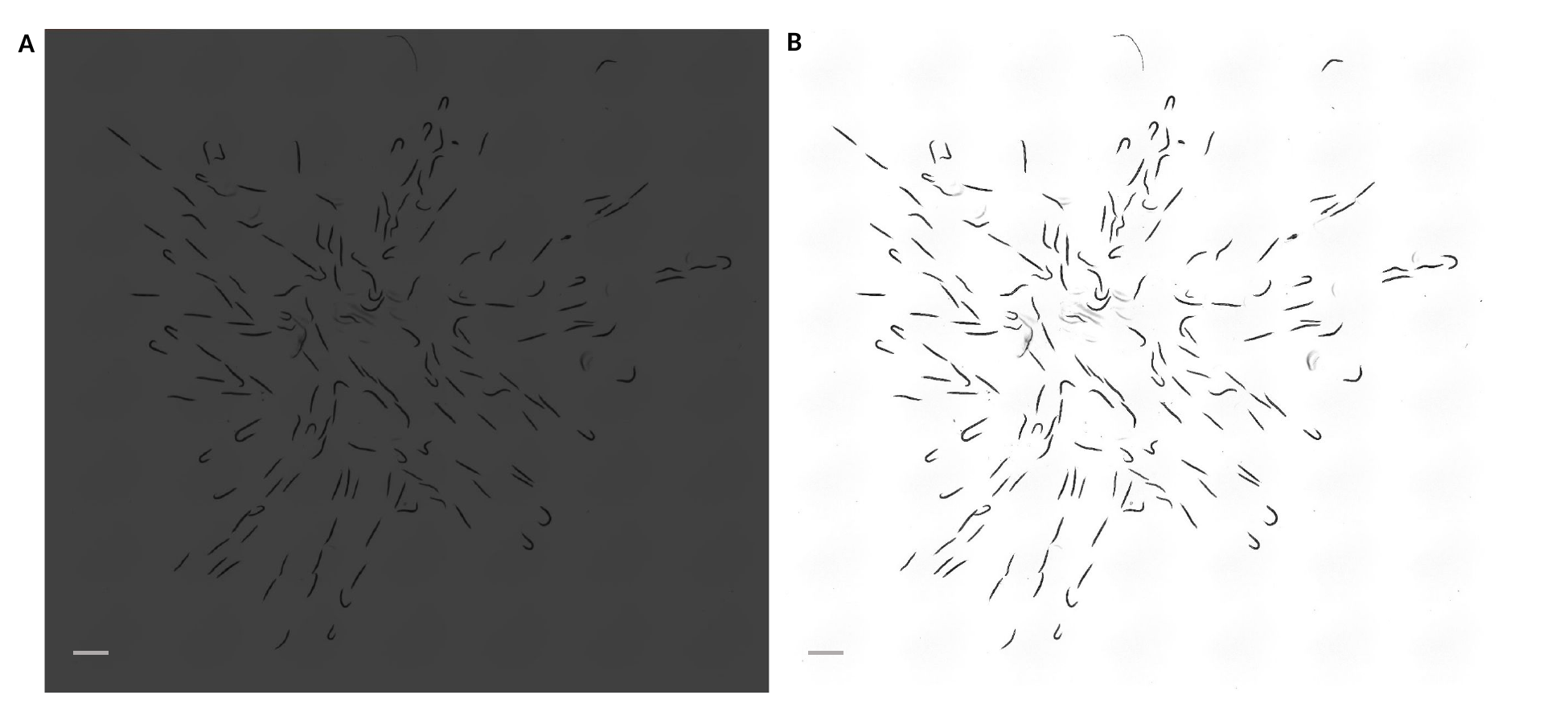

B
A

Supplement: Supplementary file 2 — Typical input image. A standard image as acquired by the microscope (A) and after grayscaling (B). Scale bar 1 mm. Both images were compressed for their addition to the paper, but their original size was 1.1 GB. The resolution was 72 dpi with 16 bit depth, and the dimension was 17,610 × 16,116. (PPTX 355 kb) [file 12915_2017_477_MOESM2_ESM.pptx]

## Slide 1
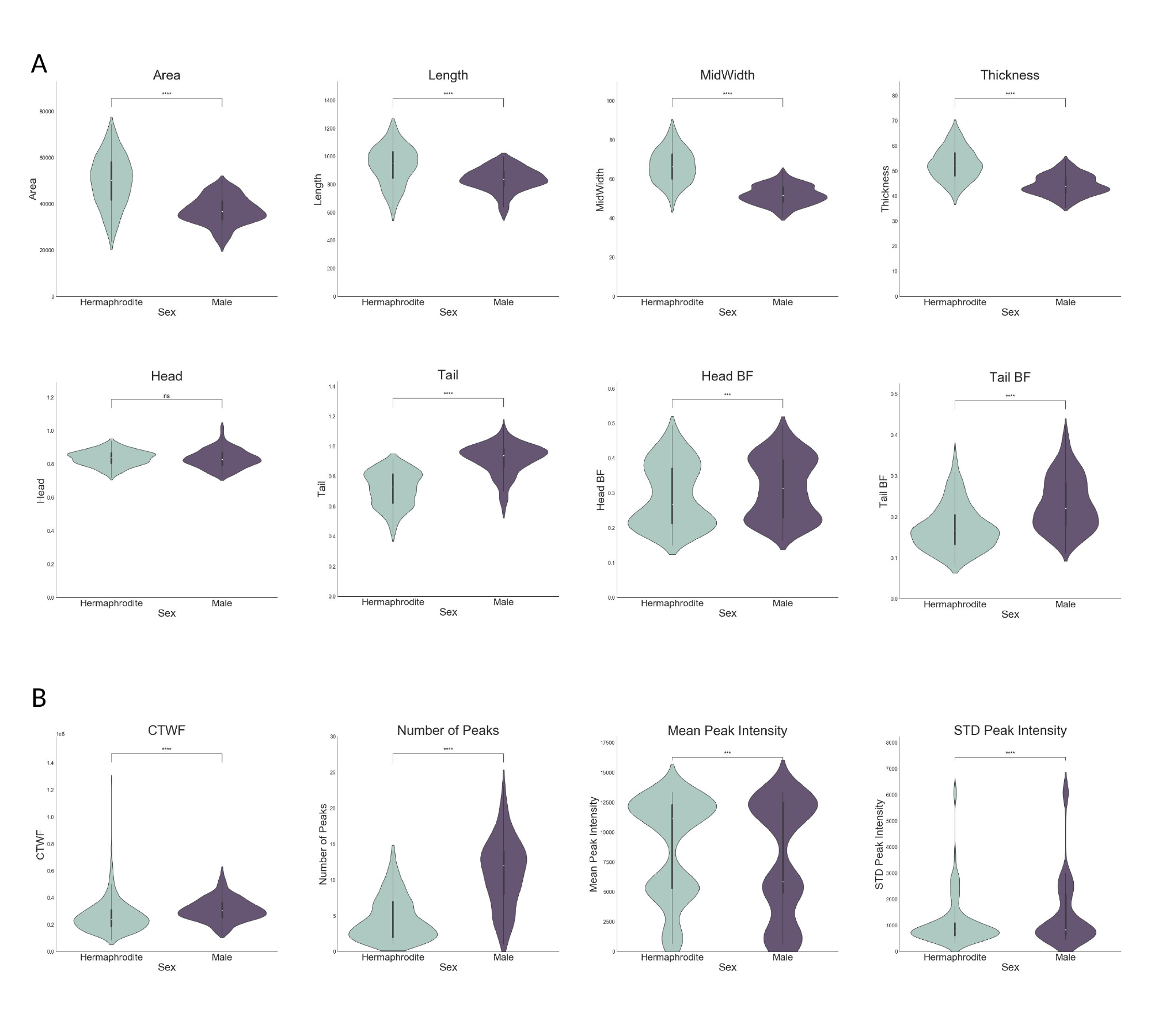

A
B

Supplement: Supplementary file 4 — Features used to establish worm masculinity. Violin plots show the morphological features (A) and fluorescent features (B) used to determine the masculinity of him-5; [tph-1p::GFP] worms. A total of 545 pre-labeled worms of each sex were used for analysis (****p < 10–4, ***p < 10–3, *p < 0.05, two-tailed t test after α = 0.01 trimming to exclude extreme outliers with false discovery rate (FDR) correction for multiple comparisons). Only features that were significantly distinct and had plausible theoretical justification to differentiate between sexes were used for sex phenotype prediction. As can be seen in this figure, males and hermaphrodites differ in some features but not in every feature examined. (PPTX 597 kb) [file 12915_2017_477_MOESM4_ESM.pptx]

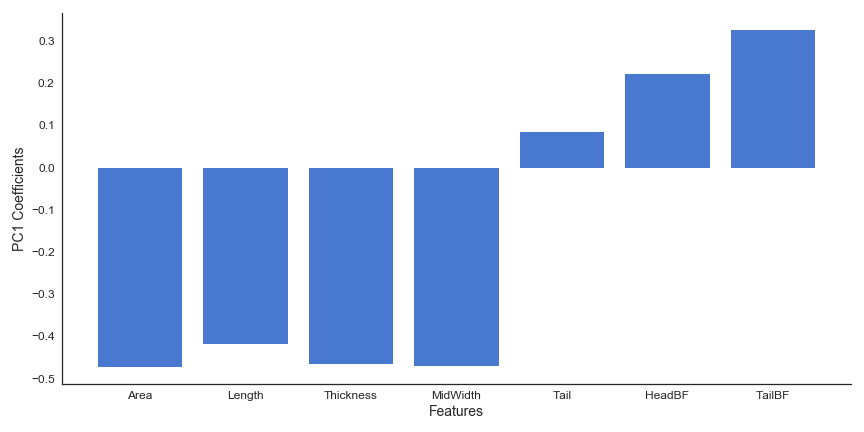

Supplement: Supplementary file 5 — Features contribution. Demonstrates the relative contribution of each feature to the separation across the first component of the PCA in Fig. 3. (PNG 13 kb) [file 12915_2017_477_MOESM5_ESM.png]

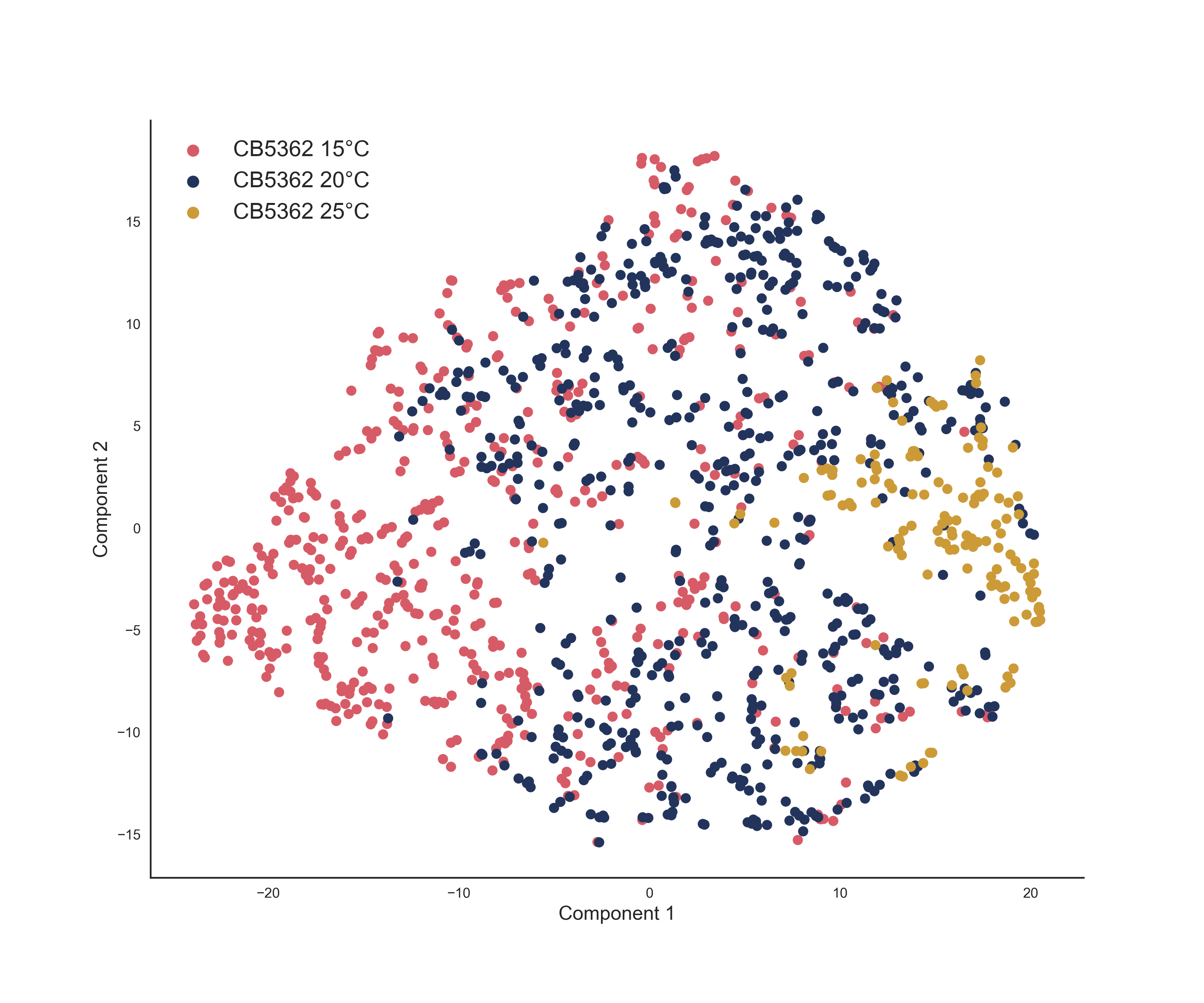

Supplement: Supplementary file 6 — t-SNE visualizing the effect of temperature on the sexual phenotype. The same assay presented in Fig. 3 analyzed with t-SNE instead of PCA. (PNG 692 kb) [file 12915_2017_477_MOESM6_ESM.png]

## Slide 1
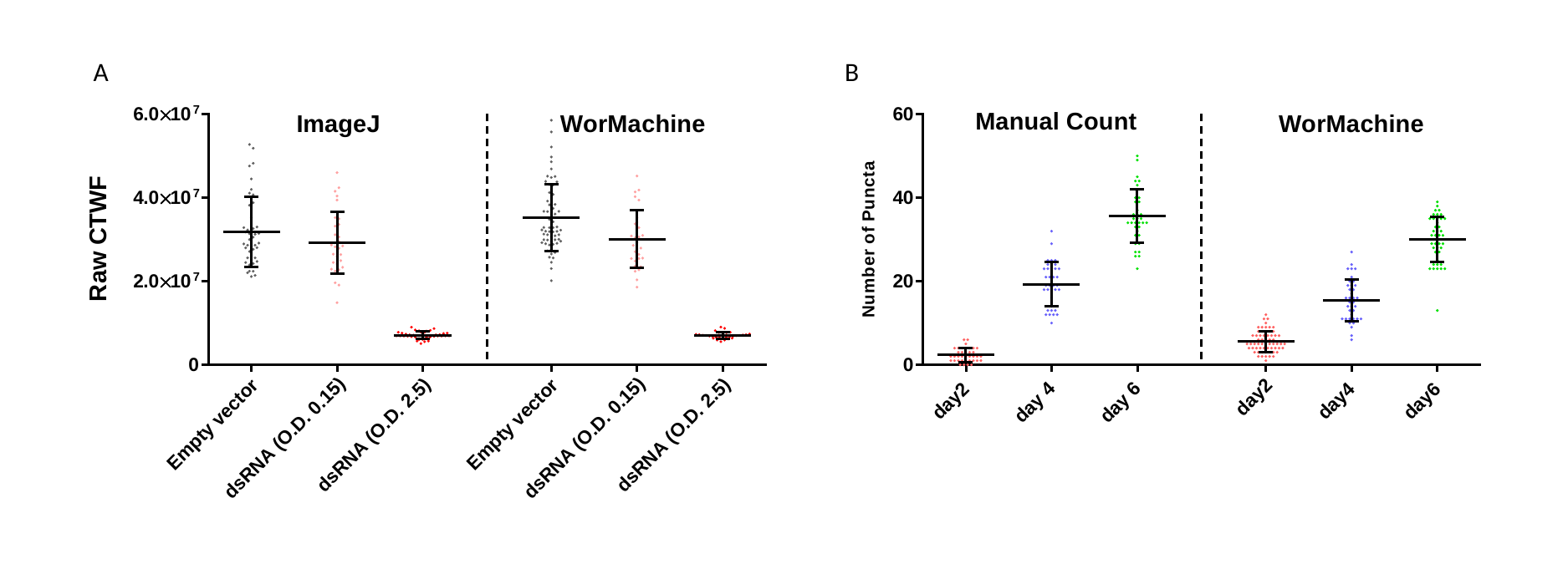

A
B

Supplement: Supplementary file 7 — Comparisons between scoring of different phenotypes using WorMachine and alternative scoring methods. A. CTWF measurements, taken either manually using ImageJ or automatically by WorMachine. B. Number of puncta, counted manually, or automatically by WorMachine. (PPTX 296 kb) [file 12915_2017_477_MOESM7_ESM.pptx]

## Slide 1
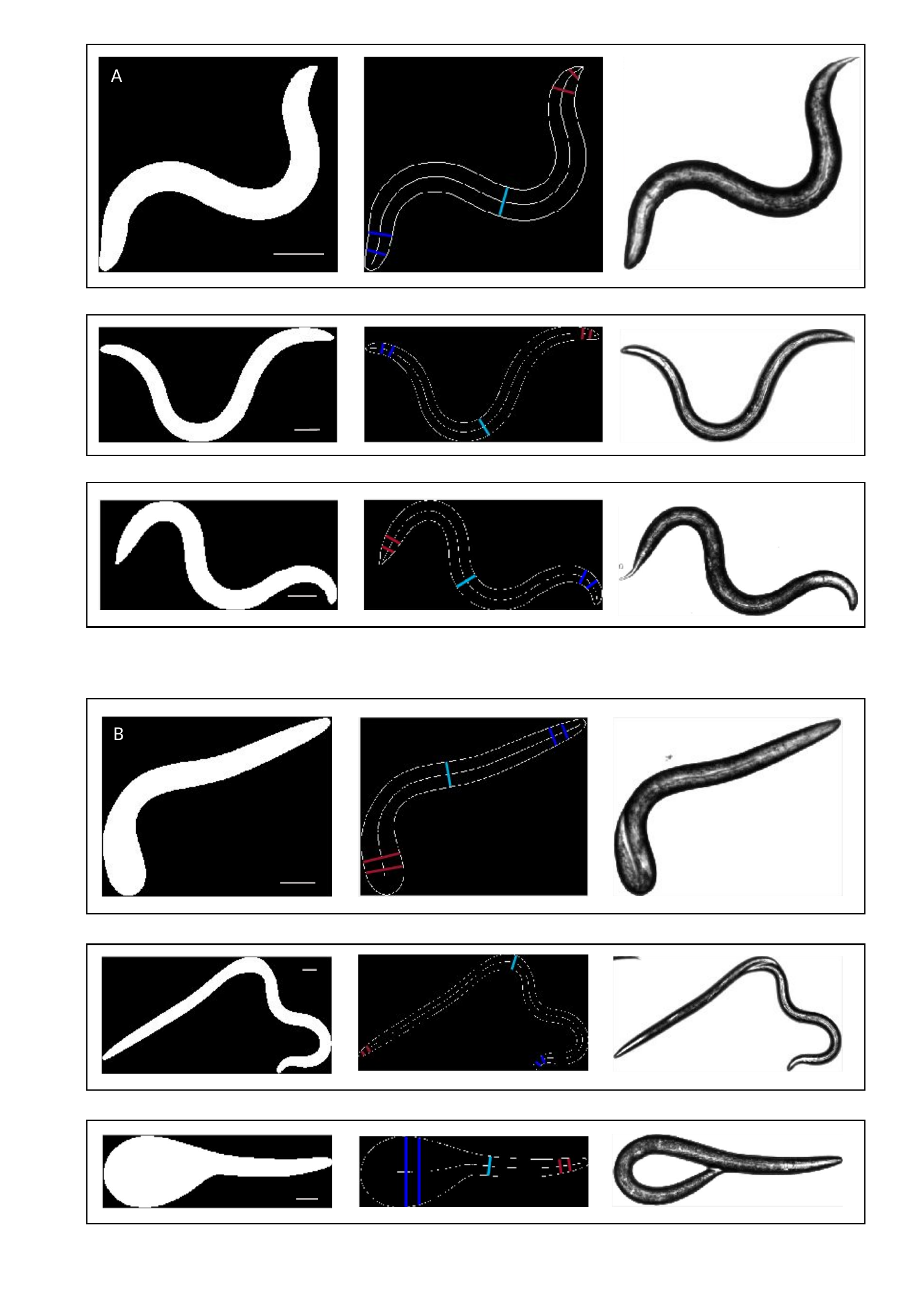

A
B

Supplement: Supplementary file 8 — Various worm images demonstrating WorMachine’s capabilities and limitations. A. Images show the software’s ability to skeletonize and analyze worms in a variety of postures. B. The software is limited in identifying worms that touch another worm or any point on their own body. These are flagged as faulty. Scale bar indicates 50 μm. (PPTX 204 kb) [file 12915_2017_477_MOESM8_ESM.pptx]
