# Supplementary material for: WorMachine: machine learning-based phenotypic analysis tool for worms
Source: BMC Biol. 2018 Jan 16;16:8. doi: 10.1186/s12915-017-0477-0 (PMC5769209; doi:10.1186/s12915-017-0477-0)
Supplement: Supplementary file 3 — Reliability of worm identification. Results showed percentage of worm retention from initial object count. This demonstrates that once objects are identified by the Image Processor, they are likely to be identified as a “real worm” rather than be removed as noise. (DOCX 11 kb) [file 12915_2017_477_MOESM3_ESM.docx]

| Independently replicated experiments | 1 | 2 | 3 |
| --- | --- | --- | --- |
| Initial object count | 323 | 488 | 739 |
| Objects found in image processor | 100.00% | 100.00% | 100.00% |
| Worms identified in image processor | 95.95% | 96.12% | 97.03% |
| Worm count after analysis and manual removal | 87.02% | 84.19% | 85.95% |

**Additional file 3: Table S1. Reliability of worm identification.** Results showed high percentage of worm retention from initial object count. This demonstrates that once objects are identified by the image processor, they are very likely to be identified as a “real worm”, rather than be removed as noise.
